# Lifestyle Habits and Exposure to BPA and Phthalates in Women of Childbearing Age from Northern Italy: A Pilot Study

**DOI:** 10.3390/ijerph18189710

**Published:** 2021-09-15

**Authors:** Ilaria Di Napoli, Sara Tagliaferri, Eduardo Sommella, Emanuela Salviati, Debora Porri, Benedetta Raspini, Hellas Cena, Pietro Campiglia, Cinzia La Rocca, Rosa Maria Cerbo, Rachele De Giuseppe

**Affiliations:** 1 Laboratory of Dietetics and Clinical Nutrition, Department of Public Health, Experimental and Forensic Medicine, University of Pavia, 27100 Pavia, Italy; ilaria.dinapoli@unipv.it (I.D.N.); debora.porri01@universitadipavia.it (D.P.); benedetta.raspini@unipv.it (B.R.); rachele.degiuseppe@unipv.it (R.D.G.); 2 Department of Medicine and Surgery, University of Parma, 43126 Parma, Italy; sara.tagliaferri@unipr.it; 3 Department of Pharmacy, University of Salerno, Fisciano, 84084 Salerno, Italy; esommella@unisa.it (E.S.); esalviati@unisa.it (E.S.); pcampiglia@unisa.it (P.C.); 4 Clinical Nutrition and Dietetics Operative Unit, Internal Medicine and Endocrinology, Istituti Clinici Scientifici Maugeri IRCCS, 27100 Pavia, Italy; 5 Gender Prevention and Health Section, Center for Gender-Specific Medicine, Istituto Superiore di Sanità, 00161 Roma, Italy; cinzia.larocca@iss.it; 6 Neonatal Unit and Neonatal Intensive Care Unit, Fondazione IRCCS Policlinico San Matteo, 27100 Pavia, Italy; rm.cerbo@smatteo.pv.it

**Keywords:** endocrine-disrupting chemicals, bisphenol A, phthalates, lifestyle, eating habits

## Abstract

*Background:* Endocrine-disrupting chemicals (EDCs) are compounds that interfere with aspects of hormonal signaling. Considerable attention has been paid to their biological effects especially in women of childbearing age or during pregnancy as EDCs have been reported to cross the placenta becoming concentrated in the fetus’ circulation. Lifestyle habits, daily consumption of packaged foods and use of healthcare/cosmetic products are associated with increased EDCs levels. This cross-sectional research examined the EDCs levels and the lifestyle determinants of EDC exposure in a cohort of reproductive-age women from Northern Italy. *Methods:* Forty-five women (median age: 36, IQR: 30–38) were evaluated for urinary bisphenol A (BPA) and phthalates levels and also studied for EDCs’ major determinants of daily exposure; food frequency/dietary, physical activity, smoking habits and weight status. *Results:* Although 100% of women seemed to have been exposed to common sources of EDCs, they reported a healthy lifestyle. The multivariable model described a positive and significant association between consumption of sauces/dressings in plastic containers and monoethyl phthalate exposure (*p* = 0.037). *Conclusions:* Since reproductive age encompasses a critical window for future health and functioning of the “mothers-to-be” and their children, future studies on prenatal dietary BPA and phthalate exposure and the role of consumer product choices in reducing such exposure are recommended.

## 1. Introduction

Endocrine-disrupting chemicals (EDCs) are compounds that interfere with several aspects of endogenous hormonal signaling, affecting not only hormone production, release and transport but also cellular metabolism, binding action and elimination [1]. This group of chemical compounds includes synthetic chemicals such as industrial solvents, plastics, plasticizers, fungicides, pesticides, heavy metals and pharmaceutical agents [2].

In recent decades, urinary concentrations of EDCs or their metabolites have been measured in many biomonitoring programs and used as exposure biomarkers for the global general population [2,3,4,5,6]. These compounds have recently been banned or restricted in certain products—sometimes not completely and with differences among countries—due to their suspected toxicity and endocrine disruptive role [7] with a negative impact on human health. In particular, it has been shown that EDCs, such as bisphenol A (BPA) and phthalates, can mimic or interfere with estrogenic, androgenic, glucocorticoid, thyroid and insulin signaling pathways, leading to a variety of tissue-specific effects, primarily in adipose tissue, reproductive organs and the thyroid but also in the liver and pancreas [8].

For instance, BPA is a chemical compound with structural similarities to 17ß- estradiol [9]. It has been reported that excessive exposure causes reproductive and developmental issues [10], including damage to the pituitary–thyroid axis and alterations in thyroid hormone levels [11], increased risk of type 2 diabetes mellitus [12], impaired neuropsychological development [13] and increased risk of low birth weight and adverse birth outcomes [14,15]. Phthalates are also known as a class of EDCs with estrogenic and anti-androgenic effects [16]. Phthalates or their metabolites mainly affect the human endocrine and reproductive system, leading to developmental and reproductive impairment [17], growth retardation [18,19], neurodevelopmental problems [20], allergies and asthma [21].

Bisphenol A (BPA) and phthalates are also ubiquitous in the environment as they are primary chemical structure blocks in the plastics industry [22,23,24]. BPA is mainly used as a monomer in the production of light-weight polycarbonate plastics and epoxy resins, which are found commonly in a variety of consumer goods, such as reusable storage containers, tableware, water bottles, plastic wrap and microwave ovenware [25,26]; phthalates are usually present in products such as pharmaceuticals, cosmetics and personal care products [27].

Several studies have demonstrated the presence of phthalates and BPA in the human body, and their effects on human health are increasingly gaining more attention globally [28,29]; currently, different studies have shown that some lifestyle habits such as diet, eating habits, daily consumption of packaged foods and daily use of healthcare or cosmetic products are associated with phthalate metabolites and BPA levels [16,30,31] in the human body [32,33].

In recent years, considerable attention has been paid by the medical-scientific community to the biological effects of EDCs; such health warnings should not be underestimated, especially when dealing with women of childbearing age or pregnant ones. Nowadays, it is widely accepted that events occurring in the early stages of life play a fundamental role in fostering the development of chronic diseases throughout life, suggesting that the “maternal environment” deeply impacts the life of the offspring [34]. This critical period, known as “the first 1000 days”, begins at the time of conception and continues until two years of age [34]. This also applies to the increased exposure to EDCs that can have long-lasting health effects during fetal development and childhood, as there are times when hormones regulate organ formation and maturation [35,36]. Early exposures have been linked to developmental abnormalities and may increase the risk of disease later in life. Of note, several EDCs have been reported to cross the placenta and concentrate in the fetus’ circulation; other EDCs can be transferred from mother to infant through breast milk [35,36].

Therefore, given the wide exposure to EDCs according to daily lifestyles and the influence that the lifestyle of women of childbearing age exerts both on their health and potentially on the future health of offspring, this cross-sectional study aimed to study BPA and phthalate levels and the lifestyle determinants of EDC exposure in a cohort of women of childbearing age.

## 2. Materials and Methods

The present research is an ancillary study of the longitudinal A.MA.MI (Alimentazione MAmma e bambino nei primi MIlle giorni) Project (clinicalTrials.gov identifier: NCT04122612) [34], investigating the correlation between the infant gut microbiota composition and maternal and infant lifestyle and environmental factors, including EDCs, from conception to the first year of life. The study was conducted according to the guidelines of the Declaration of Helsinki and approved by the Ethics Committee of the Fondazione IRCCS Policlinico S. Matteo of Pavia (protocol number: 20180022618, 12 June 2018; addendum, protocol number: 20190068597, 1 August 2019), and it was conducted on a group of mother–infant pairs referred to the Neonatal Unit, Fondazione IRCCS Policlinico San Matteo, (Pavia, Italy), according to the good clinical practice guidelines. Written informed consent was provided. The Human EC of Fondazione IRCCS Policlinico S. Matteo of Pavia approved this procedure after ascertaining its compliance with the dictates of the Declaration of Helsinki (IV Adaptation).

This cross-sectional ancillary study included the forty-five women previously enrolled in the A.MA.MI Project and evaluated for EDC exposure at 12 months after delivery [34]. The study aimed at evaluating the major determinants of BPA and phthalate exposure in a cohort of women of childbearing age and the association between EDC levels and lifestyle habits.

EDCs were measured in single-spot urinary samples; in particular, we assessed levels of BPA; metabolite of diethyl phthalate (DEP), named monoethyl phthalate (MEP); mono isobutyl-phthalate (MIbP) as a metabolite of -n-butyl phthalate (DnBP); metabolites of DEHP, such as mono (2-ethyl-5-hydroxylhexyl) phthalate (MEHHP) and mono (2-ethylhexyl) phthalate (MEHP); mono benzyl phthalate (MBzP) and metabolite of benzyl butyl phthalate (BBP). Women were also investigated for (i) medical history and demographic data, including occupation (housewife vs. other employment), level of education (tertiary education vs. non-tertiary education) and presence of chronic diseases (yes vs. no); (ii) major determinants of daily exposure to EDCs; (iii) lifestyle habits, including food consumption frequency/eating habits, physical activity level and smoking habits; and (iv) anthropometric parameters.

### 2.1. Evaluation of Determinants of Daily Exposure to Phthalates and BPA

The determinants of BPA and phthalate exposure were recorded through a structured telephone interview conducted by qualified personnel. In particular, the questions were extrapolated from the questionnaire designed in the LIFE PERSUADED (“Phthalates and bisphenol A biomonitoring in Italian mother–child pairs: link between exposure and juvenile diseases”) project [37], funded by the European Commission (under grant agreement LIFE13 ENV/IT/000482). Briefly, the LIFE PERSUADED questionnaire evaluated the association between demographic and lifestyle variables potentially related to DEHP/BPA exposure and internal levels in children and their mothers [37].

In this ancillary study, in collaboration with the LIFE PERSUADED project, some elements of the LIFE PERSUADED questionnaire [37] were used to collect information on environmental factors, lifestyle and food habits that exposed women to EDCs. All investigated exposure determinants are listed in Table 1.

### 2.2. Lifestyle Habits and Anthropometric Parameters’ Evaluation

Out of nine sections of a previously validated self-administered dietary questionnaire [38], two sections were utilized to investigate food consumption frequency and food habits. Note, that the questionnaire was originally drawn from one developed and validated in an Italian youth population [38] and then adapted by two dieticians to the adult population before its administration. The newly adapted version was previously tested on a sample of 24 subjects and revised accordingly [39].

The food frequency (FF) section (18 questions) evaluated the daily consumption of typical foods and drinks such as bread, pasta, cereal products, fruit and vegetables, milk and yoghurt, tea and coffee and weekly consumption of other foods such as meat and meat products, fish, eggs, cheese, legumes and sweets [38]. Alcoholic beverage intake was also investigated [38]. Each section consisted of questions including multiple choice answers with the following response categories: always, often, sometimes, never [38]. The score assigned to each response ranged from 0 to 3, with the maximum score assigned to the healthiest one and the minimum score to the least healthy one [38].

The food habit (FH) section (14 questions) was designed to investigate eating habits including breakfast consumption, the daily number of meals, fruit and vegetable intake and consumption of non-alcoholic or alcoholic drinks [38]. In this section, some questions were aimed at evaluating whether the number of portions consumed satisfied the recommendations [38]. Eight of the questions had the following response categories: always, often, sometimes, never; the score assigned to each response ranged from 0 to 3. The other six questions had instead four response categories structured in different ways, and the score ranged from 1 to 4. In both cases, the maximum score was assigned to the healthiest one and the minimum score to the least healthy one; the total score of the FH section was 56 [38].

The total score was then divided into tertiles, where the lowest one referred to “inadequate eating habits”, the medium one referred to “partially satisfactory eating habits” and the highest one referred to “satisfactory eating habits”, according to the National Dietary Guidelines [40].

Physical activity during the last 7 days was investigated by using the validated International Physical Activity Questionnaire, short-form (IPAQ; short form) [41]. The appropriate adapted Italian IPAQ version questionnaire was downloaded [42], and according to its scoring protocol, the metabolic equivalent of task (MET-min) per week was calculated as

*METs = MET level × minutes of activity × events per week*

Physical activity level was also classified as sedentary (total METs < 699), moderate (total METs range 700–2519) and high (total METs > 2520) [41].

Smoking habits were investigated, and women were classified as smokers, never smokers or former smokers.

Anthropometric parameters, including height (cm), weight (kg), hip (cm) and waist circumference (WC, cm) were measured with standardized procedures as described elsewhere [43]. Body mass index (BMI) was calculated as the ratio of weight (kg) to height (m) squared [43]. The waist-to-hip ratio (WHR) and waist (cm) to-height (cm) ratio (WHtR), good predictors of abdominal adiposity in adults, were also calculated [44].

### 2.3. Phthalate, BPA and Creatinine Level Determination

#### 2.3.1. Sample Collection and Analysis

BPA and phthalate (MEP, MIbP, MEHHP, MBzP, MEHP) levels were measured in mid-stream clean urine samples instead of in first or 24 h urine samples, according to Lee et al. [45], and creatinine concentrations were also assessed.

Briefly, urinary samples were collected into polypropylene cups for urine culture, and then women were asked to store the cups in a cool place until transportation. The urine samples were transported under refrigeration, aliquoted into tubes made from phthalate-free materials and stored at −80 °C until analysis.

BPA and phthalates levels were measured by UHPLC-MS/MS (Shimadzu, Milan, Italy). The UHPLC system consisted of two LC 30 AD pumps, a SIL 30 AC autosampler, a CTO 20 AC column oven and a CBM 20 A controller, and the system was coupled online to a triple quadrupole LCMS 8050 (Shimadzu, Kyoto, Japan) equipped with an electrospray ionization (ESI) source. Results were compared to previous data (Table S1, Supplementary Materials).

Creatinine levels were measured by routine methods on an Atellica^®^ CH Analyzer (Siemens Healthcare Diagnostics Inc., Tarrytown, NY, USA) and expressed as μmol/L.

All urinary samples were considered acceptable because the creatinine concentrations were within the range of 0.3–3.0 g/L [46].

#### 2.3.2. BPA and Phthalate Metabolite Extraction

Urine samples (1 mL each) were thawed on ice; samples were diluted with 1 mL of 1 M of ammonium acetate (pH 7), 4 μL of an internal standard mixture in methanol and 10 microliters of beta-glucuronidase were added. The reaction was carried out for two hours at 37 °C under gentle shaking; after two hours, the reaction was quenched with 100 μL of glacial acetic acid. The samples were subjected to solid-phase extraction employing polymeric reversed-phase cartridges (Strata X, Phenomenex, Bologna, Italy). Cartridges were conditioned with 2 mL of MeOH and equilibrated with 2 mL of H_2_O, for the washing phase, 3 mL of H_2_O was used, the cartridges were dried under vacuum for 5 min, samples were finally eluted with 3 mL of ACN. Samples were dried under nitrogen and reconstituted in 200 μL of 70% MeOH, filtered through a 0.45 μm mesh and then injected in UHPLC-MS/MS. All reagents were purchased by Merck Sigma Aldrich (Merck Sigma Aldrich, Milan, Italy). Phthalate metabolites and BPA standards were purchased by Cambridge Isotopes (Tewksbury, MA, USA).

#### 2.3.3. UHPLC-MS/MS Conditions

The separation was performed on a Kinetex Phenyl–Hexyl column with geometry (L × I.D) 50 mm × 2.1 mm, 2.6 μm (Phenomenex) employing as mobile phases: (A) 0.1% of CH_3_COOH in H_2_O and (B) 0.1% of CH_3_COOH in ACN for phthalate metabolites, while 0.1% NH_4_OH was used to enhance ionization for bisphenol detection in both phases. Phthalate metabolites were eluted with the following gradient: 0 min, 20% B, 0.1–5 min, 20–35% B, 5–7 min, 35–100% B, hold for 1 min, while BPA was eluted as follows: 0 min, 20% B, 2 min, 100% B hold for 30 s. The flow rate was set to 0.5 mL/min. The column oven was set to 40 °C, and 2 and 5 μL of extract were injected for phthalate metabolites and BPA, respectively. All additives and mobile phases were of LCMS grade and were purchased from Merck (Milan, Italy).

The ESI was operated in the negative mode. MS/MS analysis was conducted in scheduled multiple reaction monitoring (MRM), the transition was optimized by flow injection analysis of the corresponding standard compounds. Interface temperature, desolvation line temperature and heat block temperature were set to 250, 200 and 350 °C, respectively. Nebulizing gas, drying gas (N_2_) and heating gas (air) were set to 3, 10 and 10 L/min, respectively.

Repeatability was established by triplicate injections of sample and solutions at low, medium and high concentration levels of the calibration curve with the same chromatographic conditions and analyst on the same day, and within two consecutive days, results were expressed as CV% for concentration and retention time. Limits of detection (LODs) and quantification (LOQs) were calculated by the ratio between the standard deviation (SD) and analytical curve slope multiplied by 3 and 10, respectively. The LOD values (ng/mL) we calculated were the following: 0.061 for MEP, 0.733 for MIbP, 0.956 for MEHHP, 0.064 for MBzP, 0.056 for MEHP and 0.574 for BPA. The LOQ values (ng/mL) were as follows: 0.204, 2.443, 3.186, 0.212, 0.187 and 1.041, respectively, for MEP, MIbP, MEHHP, MBzP, MEHP and BPA.

Recovery was assessed by spiking a known amount of each standard at the low, medium and high concentration ranges.

### 2.4. Statistical Analysis

The normality of data distribution was assessed by the Shapiro–Wilk test. Normally, log-normally distributed and non-normalizable variables were reported as mean and SD, geometric mean (GM) and geometric SD (GSD), median and interquartile range (Q1–Q3), respectively. When urinary concentrations of EDCs were below the LOD, a value equal to LOD/2 was used.

Given the absence of outliers with an excessive influence on models with not-transformed or log-transformed variables, Pearson’s univariate correlation coefficient was always calculated as log values. To assess the overall relationship among the three highly correlated chemicals (MEHHP, MBZP and MEHP), a model of principal component analysis (PCA) was used with the following options: communality always near 0.7, overall explained variance well above 50%.

Finally, to explore major sources of exposure to EDCs, multiple regression models were performed. Each model included as dependent variables EDCs’ urinary concentrations and the score of the factor resulting from PCA and as potential predictors all the relevant covariates showing significance at the univariate analysis. Only variables with a variance inflation factor (VIF) <2 were included to exclude multicollinearity. In the BPA model, the covariates were creatinine, takeaway cooked food consumption and FF section—food frequency. In the MEP model, we included consumption of creatinine and sauces or dressings in plastic containers. In the Factor 1 model, creatinine and high-frequency use of canned (or tetra packed) food were the covariates included.

A *p*-value of 0.05 was always considered significant. All of the statistical analyses were made using the statistical software IBM SPSS Statistics 25 (IBM, Armonk, NY, USA).

## 3. Results

### 3.1. Study Population

Forty-five women of childbearing age previously enrolled in the A.MA.MI Project [34] were considered.

As shown in Table 2, 50% of women enrolled were aged less than 36 years old; overall they were healthy (12.9% of women were affected by chronic diseases such as rheumatoid arthritis, chronic migraine, hypothyroidism or healthy carrier of beta-thalassemia), with body weight and fat distribution suggestive of low metabolic and cardiovascular disease risk. In particular, 62.8% of women were normal weight [40]; 79.5% of them had WC lower than 88 cm (WC, cutoff value <88.0 cm) [40]; WHR was lower or equal to 0.85 in all subjects (WHR, cutoff value <0.85) [47] and for 55.8% of subjects, the WHtR value was lower or equal to 0.48 (WHtR cutoff value <0.5) [37]; 58.1% of women had completed tertiary education and the majority of them were employed (12.9% were housewives and 87.1% other occupations), spending 8 h/day at work.

### 3.2. Determinants of Daily Exposure to Phthalates and BPA and Lifestyle Habits

To explore determinants of daily exposure as variables potentially related to phthalates and BPA, a telephone interview extrapolating questions from the LIFE PERSUADED Project [37] was conducted. The main results are reported in Table 1. About 63.4% of women used plastic products for daily personal care (lotions, make-up, dental care, glasses, gloves), 53.7% had contact with polyvinyl chloride (PVC) in their home, work or recreational activity environments, while exposure to synthetic materials at the gym or during recreational activities of free time was observed for 17.8% of subjects.

The frequent consumption of packaged food in plastic containers or film-wrapped food, canned (or tetra packed) food was described for the entire group of women. We observed a frequency of one or more times/day for using packaged food in plastic containers or film-wrapped food in 58.1% of subjects, 38.7% used one or more times/day canned (or tetra packed) food (58.1% of the study group consumed canned food 1–3 times/week) (data not shown). Moreover, 41.9% of women consumed packaged food or pre-cooked food to be heated (46.2% of them 1–3 times/week and 38.5% 1–3 times/month, data not shown). Meanwhile, 80.6% of women frequently used cooked takeaway food, 22.6% used disposable plastics. We observed the frequent use of plastic containers for storage of cooked or raw food in 90.3% of our sample, and 71.4% used the dishwasher to clean them.

A high percentage (89.3%) of women used film to package and store food, 74.1% used plastic containers in the microwave with a frequency of one or more times/day in 75% of cases (data not shown). About half of microwave users transferred food from plastic containers to glass or ceramic dishes, while about 42% cooked or defrosted by using the original or plastic container; 48.4% of women regularly used plastic utensils for cooking.

The consumption of water from plastic bottles was observed for 64.5%, while 67.7% of women used sauces or dressings in plastic containers and 90.3% consumed packaged dressings or dressings from the original packaging.

Data on food frequency (FF) and food habits (FH), level of physical activity and smoking habits were collected using dedicated questionnaires (Turconi and IPAQ questionnaires) and a semi-structured interview (Table 3). The FF section analysis revealed that about 34% of our sample reported the healthiest food consumption frequency (third tertile: 37–42 score), about 32% had moderately healthy food frequency (second tertile: 32–36.7 score), while 34% showed less healthy ones (first tertile: 22–31 score), according to the National Dietary Guidelines [40]. Furthermore, about 63% of women enrolled were characterized by partially adequate (second tertile: 36–39 score) or satisfactory eating habits (third tertile: 40–47 score) (both categories 31.8%), while about 36.4% were in the first tertile (28–35 score), suggestive of inadequate eating habits, according to the National Dietary Guidelines [40]. The IPAQ (1386 [693–2772] METs) median value was representative of a moderate/active profile for our population, with 70.5% of women with a moderate or high level of physical activity vs. 29.5% of sedentary ones. Only 16.1% of women were smokers (Table 3).

### 3.3. Phthalate and BPA Levels

BPA and phthalates were present at detectable concentrations in all samples, except for MIbP, for which 25.5% of samples showed a level below the LOD.

The GM (GSD) of total urinary concentrations of EDCs (unadjusted, µg/L and creatinine-adjusted, µg/g creatinine) are shown in Table 4. Overall, MEP was the chemical more represented (28.68 (3.10) µg/g creatinine), the toxicant at the lowest concentration observed in urine samples was MEHP (0.93 (4.13) µg/g creatinine).

### 3.4. Principal Component Analysis

Pearson’s univariate explorative analysis was performed among all EDCs (unadjusted, µg/L), showing correlation coefficients lower than 0.6 for BPA, MEP and MIbP, while the intensity of the relationship among MEHHP, MBzP and MEHP was higher (Table 5), suggesting multiple concurrent exposures to those contaminants.

PCA performed on inter-correlated chemicals distinguished one factor (Factor 1), grouping MEHHP, MBzP and MEHP. Communality values were 0.94, 0.64 and 0.94 for MEHHP, MBzP and MEHP, respectively. The total explained variance of the model was 84.1%.

### 3.5. Univariable Models

Univariable models, considering as dependent variables urinary concentrations of BPA (Model 1), MEP (Model 2), MIbP (Model 3), the score of Factor 1 (Model 4) and as covariate creatinine levels and all items of lifestyle and eating habit questionnaires, were performed. Significant correlations are shown in Table 6. In all univariable models, we observed positive and significant correlations between chemicals and creatinine concentrations (g/L) (Model 1, r = 0.61, *p* < 0.001; Model 2, r = 0.4, *p* = 0.009; Model 3, r = 0.36, *p* = 0.016; Model 4, r = 0.75, *p* < 0.001). In Model 1, although weaker if compared to creatinine, significant relationships with takeaway food consumption and the score of the food frequency section of the Turconi questionnaire (r = 0.38, *p* = 0.04; r = −0.37, *p* = 0.01, respectively) were described. A Pearson’s coefficient value r = 0.43 (*p* = 0.016) was calculated between MEP level (Model 2) and consumption of sauces or dressings in plastic containers, and a negative and significant relationship emerged between Factor 1 and high frequency of consumption of canned (or tetra packed) food (r = −0.36, *p* = 0.04).

### 3.6. Multivariable Regression Models

To explore the major determinants of exposure to EDCs, multiple regression models, including predicting variables that were significant at the univariate approach, were performed, and the results are shown in Table 7. Multivariable analysis confirmed the creatinine level as a significant predictor of urinary concentrations of BPA and Factor 1 (β = 0.63, *p* < 0.001; β = 0.71, *p* < 0.001, respectively), while the relationship with MEP did not reach statistical significance (β = 0.35, *p* = 0.06). The association between BPA concentrations and takeaway or cooked food consumption was not confirmed by the model, even though the *p*-value was close to statistical significance (β = 0.27, *p* = 0.06). The habit of consuming sauces or dressings in plastic containers was described as an independent factor contributing to exposure to MEP (β = 0.37, *p* = 0.037).

## 4. Discussion

The present ancillary study investigated for the first time lifestyle habits as potential determinants of exposure to BPA and phthalates in a group of women of childbearing age from Northern Italy, using dedicated questionnaires [38,41], interviews and urinary EDC concentration measures. Our sample population was characterized by overall healthy lifestyle habits including a moderate–high level of physical activity and moderately healthy and adequate eating habits. On the other hand, a high percentage of women enrolled seemed potentially exposed to common sources of BPA and phthalates, as 100% of study participants were used to consuming and storing food in plastic containers, film-wrapped food and canned food and exposed plastic containers to high temperature. We found that consumption of sauces or dressings in plastic containers was an independent determinant of MEP urinary concentration and that creatinine level was associated with increased levels of BPA and the score of Factor 1 resulting from PCA of chemicals highly correlated among themselves (MEHHP, MBzP and MEHP).

The adapted version of the Turconi et al. questionnaire [38] and the IPAQ questionnaire [41] allowed us to estimate a prevalence of rather healthy lifestyle habits for more than 60% of the women we enrolled. The study of Bedrick et al. [48] confirmed the presence of two dominant dietary patterns in a cohort of midwestern women of reproductive age. In particular, according to a food frequency assessment, one pattern was defined as healthier and the other one less healthy, with prevalence values similar to those we observed in the present research and falling in the range of previous studies [48]. Some studies described different eating habits of women of childbearing age, finding that they did not meet recommendations for multiple nutrients or that there were no differences compared with the dietary behaviors of women of the same age planning a future pregnancy and potentially interested in improving health-related behaviors [49].

The moderate–high level of physical activity we observed in about 70% of the women enrolled appears supported by similar healthy lifestyle habits described by Bedrick et al. [48] in a cohort of women of childbearing age.

Even if governments and public health agencies have made great efforts to reduce tobacco use among women of childbearing age, we estimated a 16.1% prevalence of smoking, which is in line with data from peer-reviewed literature reporting a 20.1% smoking prevalence among non-pregnant women of childbearing age [50].

To explore potential sources of exposure to BPA and phthalates, we interviewed participants using some questions extrapolated from the questionnaire designed in the LIFE-PERSUADED Project [37], aiming at evaluating the use of several products of daily life containing plasticizers associated with BPA and DEHP metabolite exposure in mothers and children [51].

The interview appeared specific and particularly suitable in exploring BPA and phthalate exposures. Notably, it investigates the use of personal care products, the presence of PVC in the home/working environments, habitual consumption of food within plastic packaging and regular use of plastic containers to store and cook food and beverages. Throughout the interview, we were able to depict the potential EDCs exposure profile of our sample, finding prevalent patterns of behavior. More than 60% regularly used plastic products for daily personal care (lotions, make-up, dental care, glasses, gloves), in line with the prevalence of 84.2% for weekly use of dermal oil and cream and 59.6% of weekly use of cosmetics estimated in a cohort of healthy Spanish women (18–50 years old) by Duran et al. [52]. Food contact materials represent another potential source of exposure to BPA phthalates. We found that almost all participants consumed pre-prepared food in plastic containers or film-wrapped (or canned) food and about 90% frequently used plastic containers or film for storage of food, with more than 70% of subjects microwaving plastic containers or cleaning them through the dishwasher. Finally, water consumption from plastic bottles was observed in 64.5% of women, and nearly 70% of the women consumed sauces or dressings in plastic containers.

These findings are not surprising, given that there have been increasing public concerns in recent years about the widespread use of consumer products containing EDCs [52]. Furthermore, several studies reporting the common consumption of this class of products among the general population have been published [26,53,54,55]. In this framework, the LIFE PERSUADED Project used its biomonitoring data to associate exposure and determinants to produce a brochure targeting the general population to promote healthy lifestyles (“10 practical tips to limit the exposure to plasticizers in children and adults”) [56].

We measured MEP, MEHHP, MBzP, MEHP and BPA urinary concentrations finding that selected EDCs were present at detectable concentrations in 100% of samples, except for MIbP, overall suggesting widespread exposure to these compounds in a cohort of healthy women of reproductive age from Northern Italy. To date, there are no studies that have examined a similar set of EDCs in Northern Italy investigating potential sources of exposure in a population of women of childbearing age. The ubiquitous presence of EDCs we observed appears consistent with previous studies in North America, Asia and Europe, showing detectable urinary BPA and phthalate levels in over 90% of those general populations and cohorts of European women of childbearing age and the general population [57,58,59,60,61,62].

The unadjusted and creatinine-adjusted urinary geometric means (geometric standard deviation) of BPA measured in our population was 3.34 (1.69) µg/L and 4.18 (1.84) µg/g creatinine, respectively, higher but in the same range of concentrations described by a recent meta-analysis of cross-sectional studies considering adult women [63]. In particular, the estimated median BPA urinary concentrations were 1.71 [0.23–3.18] μg/L and 1.65 [0.40–2.90] μg/g, unadjusted and creatinine-adjusted median values, respectively.

Regarding phthalate metabolite urine concentrations measured in our population, only MIbP levels were lower than the LOD in 25.5% of samples, possibly reflecting a lower exposure if compared to other chemicals. MEP, MEHHP, MBzP and MEHP were detectable, and overall, the GM we calculated appeared in the same order of magnitude of urinary amounts of the National Health and Nutrition Examination Survey study (NHANES), including data from the non-pregnant population of women aged 18–40, except for MEP level, which was lower in our participants (GM: 22.52 µg/L and GM: 152.9 µg/L, present study and NHANES, respectively) [64]. Even considering populations not limited to childbearing age (e.g., general population or pregnant women), concentrations of phthalate metabolites in the present cohort were overlapping data from previous studies conducted worldwide [57,58,59,60,61,62,63], supporting the ubiquitous exposure to those compounds (Table S1, Supplementary Materials).

Although EDC threshold levels that define a potential health risk have not been defined universally, cutoffs derived by human biomonitoring (HBM) of BPA, MEP, MEHHP, MBzP, MEHP and MIbP in urine have been suggested as reference values for humans to contextualize the exposure level of different populations [65,66,67,68]. Noteworthily, the urinary concentrations of BPA and phthalate metabolites in our study were far below the reference HBM values for the general population estimated by biomonitoring data. However, some recent studies in rat fetuses [69,70] have shown that maternal exposure to very low doses of BPA (2.5 µg/kg/day) was able to alter hepatic lipid metabolism and metabolic pathways important for the proper development and functioning of the brain. Furthermore, other studies [71,72] have shown that gestational exposure to BPA at low doses was able to cause cognitive deficits and influence the development of circadian centers in the offspring of rats and mice.

BPA and selected phthalates are ubiquitous; furthermore, MEHHP and MEHP are both metabolites of the same parent compound (DEHP) and both, together with MBzP, are highly correlated themselves. Thus, due to multicollinearity among them, it was not possible to distinguish the effect of a single substance. Therefore, we performed a PCA model, finding a common factor explained by MBzP, MEHHP and MEHP.

According to univariable models, creatinine concentration was positively associated with detectable BPA, MEP and MIbP concentrations and Factor 1 resulting from PCA. Among potential contributors to urinary EDC levels, we found that BPA was significantly associated with increased takeaway food consumption and worst FF score [38], and the use of sauces or dressings in plastic containers was significantly associated with MEP levels. The multivariate analysis confirmed the consumption of sauces or dressings in plastic containers as a predictor of MEP, and creatinine concentration as an independent determinant of BPA, MEP and Factor 1. The finding of the association between MEP and dressings with plastic-packaging use appears in contrast with the study by Serrano et al. [53], who did not describe a significant relationship between MEP levels and oils, butter, lard and shortening in a group of pregnant women from the U.S. However, the potential sources of exposure were only in part overlapping, and differences were present in the populations considered.

The relationship between the habit to consume takeaway pre-prepared food and urinary BPA did not reach statistical significance, probably caused by the small sample size of our study. However, Kim et al. [73] observed a urinary BPA decrease by 53% in a cohort of mothers who were on abstinence from fast food, delivery foods and foods and drinks in cans and plastic containers, suggesting the potential role of this dietary behavior as a contributing factor of urinary BPA.

Several limitations should be considered. First, as the present research is an auxiliary and pilot study, no sample size estimate was made, including only a small number of women of childbearing age. Enlarging the sample size would be appropriate to confirm the relationships we have described. Second, as the women in the study were from Northern Italy, results cannot be generalized. Third, as our study was cross-sectional, we cannot make causal inferences between lifestyle variables and urinary EDC concentrations. In this context, the results should be considered preliminary, and further studies are needed to confirm them.

Last, the data of the urine samples were compared with the lifestyle habits that emerged from the interview and questionnaires relating to the sampling period. However, the interview and questionnaires may be considered too general to reflect the EDC exposure habits immediately surrounding urine sampling.

Noteworthily, we also report several strengths. First, the investigation of food and eating habits together with the regular exposure to food-contact materials using a simple questionnaire and interview appears to be a suitable method to assess determinants of EDC exposure. Second, to the best of our knowledge, this preliminary study is among the first to include only reproductive-age women from Northern Italy aiming at exploring potential determinants of EDCs exposure. Third, the present research combined the assessment of urinary BPA and phthalates with the evaluation of eating habits and daily exposure to EDCs, using questionnaires designed for the Italian population. Last, we used principal component analysis in the context of co-exposure to multiple compounds, which allowed us to identify a factor explained by highly related chemicals.

## 5. Conclusions

In summary, we found that in a cohort of reproductive-age women from Northern Italy, overall characterized by a healthy lifestyle, including dietary and physical activity habits, there was widespread use of consumer products containing EDCs. A multivariable model described the consumption of sauces and dressings in plastic containers as a significant contributing factor to MEP exposure. Since reproductive age encompasses a critical window for future health and functioning of the “mother-to-be” and their children, future studies on prenatal dietary BPA and phthalate exposure and the role of consumer product choices in reducing such exposure are recommended.

## Figures and Tables

**Table 1:** Phthalate and BPA exposure determinants. Phthalate and BPA determinants of exposure were explored through a structured telephone interview extrapolating questions from the LIFE PERSUADED (“Phthalates and bisphenol A biomonitoring in Italian mother–child pairs: link between exposure and juvenile diseases”) Project questionnaire [37]. Data are presented as the frequency of subjects reporting the lifestyle habits listed in the table and expressed as percentages.

Determinants of Exposure	Percentage
Use of plastic products for daily personal care (lotions, make-up, dental care, glasses, gloves)	63.4%
Presence of PVC in home, working and recreational environments	53.7%
Presence of synthetic material at the gym or during recreational activities of free time	17.8%
Consumption of packaged food in plastic containers or film-wrapped food, canned (or tetra packed) food	100%
Consumption of packaged food or pre-cooked food to heat	41.9%
Cooked takeaway food consumption	80.6%
Disposable plastic use	22.6%
Frequent use of plastic containers for storage of cooked or raw food	90.3%
Use of dishwasher to clean plastic containers	71.4%
Use of film-wrap to package and store food for consumption	89.3%
Use of plastic containers in the microwave to cook or defrost food	77.4%
Transfer of food from a plastic container to glass or ceramic dish to cook or defrost by microwave	58.3%
Regular use of plastic utensils for cooking	48.4%
Consumption of water from plastic bottles	64.5%
Consumption of sauces or dressings in plastic containers	67.7%
Packaged dressings or condiments from the original packaging	90.3%

Legend. PVC, polyvinyl chloride.

**Table 2:** General characteristics of women included in the study (*n* = 45). Data were reported as mean ± standard deviation (SD) or median and interquartile range [Q1–Q3] or frequency of subjects expressed as a percentage (%).

Variables (Cutoff Value or Reference Interval)	Mean ± SD; Median [Q1–Q3]; %
Age (years)	36 [30,31,32,33,34,35,36,37,38]
BMI (kg/m^2^) Normal weight (18.5 ≤ BMI < 25) [40] Overweight (25 ≤ BMI < 30) [40] Obesity (BMI ≥ 30) [40]	22.4 ± 4.2
WC (<88 cm)	77.9 ± 9.5
WHR (<0.85)	0.49 ± 0.08
WHtR (<0.5)	0.48 ± 0.07
Subjects with chronic morbidities	12.9%
*Time spent at the workplace*	
≤8 h/day	73.9%
>8 h/day	26.1%
*Job type*	
Housewife	12.9%
Other	87.1%
*Level of education*	
<University	41.9%
≥University	58.1%

Legend. BMI, body mass index; WC, waist circumference; WHR, waist to hip ratio; WHtR, waist-to-height ratio.

**Table 3:** Lifestyle habits. Data were reported as mean ± standard deviation (SD), median and interquartile range [Q1–Q3] or frequency of subjects expressed as number (n) and percentage (%).

Lifestyle Variables	Mean ± SD; Median [Q1–Q3]; %
*FF section—food frequency (score)*	33.2 ± 5.3
Less healthy, *n* (%)	15 (34.1)
Moderately healthy, *n* (%)	14 (31.8)
Healthiest, *n* (%)	15 (34.1)
*FH section—food habits (score)*	37.6 ± 4.2
Inadequate eating habits, *n* (%)	16 (36.4)
Partially satisfactory eating habits, *n* (%)	14 (31.8)
Satisfactory eating habits, *n* (%)	14 (31.8)
*Physical activity level—IPAQ (METs)*	1386 [693–2772]
Sedentary (total METs <699, MET-min), *n* (%)	13 (29.5)
Moderate (total METs: 700–2519, MET-min), *n* (%)	15 (34.1)
High (total METs >2520, MET-min), *n* (%)	16 (36.4)
*Smoking habit*	
Smokers	16.1%
Non-smokers	83.9%

Legend. FF, food frequency section [38]; FH, food habits section [38]; IPAQ, International Physical Activity Questionnaire [41]; MET, metabolic equivalent of task.

**Table 4:** Urinary concentrations of phthalates and BPA. Data are presented as geometric mean (GM) and standard geometric deviation (GSD).

EDCs	Unadjusted (µg/L)	Creatinine-Adjusted (µg/g Creatinine)
	GM [GSD]	
BPA	3.34 [1.69]	4.18 [1.84]
MEP	22.52 [3.30]	28.68 [3.10]
MIbP	2.70 [5.00]	3.44 [4.55]
MEHHP	4.17 [2.49]	5.26 [1.87]
MBzP	2.05 [2.59]	2.56 [2.12]
MEHP	0.73 [5.73]	0.93 [4.13]

Legend. BPA, bisphenol A; MEP, monoethyl phthalate; MIbP, mono isobutyl-phthalate; MEHHP, mono (2-ethyl-5-hydroxylhexyl) phthalate; MBzP, mono benzyl phthalate; MEHP, mono (2-ethylhexyl) phthalate.

**Table 5:** Pearson’s correlation coefficients among phthalates and BPA.

	BPA (µg/L)	MEP (µg/L)	MIbP (µg/L)	MEHHP (µg/L)	MBzP (µg/L)	MEHP (µg/L)
**BPA (µg/L)**	r = 1	r = 0.14	r = 0.26	**r = 0.37**	**r = 0.36**	**r = 0.37**
**MEP (µg/L)**	r = 0.14	r = 1	r = 0.12	**r = 0.45**	**r = 0.39**	**r = 0.45**
**MIbP (µg/L)**	r = 0.26	r = 0.12	r = 1	r = 0.29	**r = 0.36**	r = 0.29
**MEHHP (µg/L)**	**r = 0.37**	**r =0.45**	r = 0.29	r = 1	**r = 0.63**	**r = 1**
**MBzP (µg/L)**	**r = 0.36**	**r = 0.34**	**r = 0.36**	**r = 0.63**	r = 1	**r = 0.63**
**MEHP (µg/L)**	**r = 0.37**	**r = 0.45**	r = 0.29	**r = 1**	**r = 0.63**	r = 1

Legend. r, Pearson’s correlation coefficient; statistically significant relationships are in bold (*p* < 0.05). BPA, bisphenol A; MEP, monoethyl phthalate; MIbP, mono isobutyl-phthalate; MEHHP, mono (2-ethyl-5-hydroxylhexyl) phthalate; MBzP, mono benzyl phthalate; MEHP, mono (2-ethylhexyl) phthalate.

**Table 6:** Pearson’s correlation analysis (univariable models).

Model	Variable	r	*p*
Model 1	Creatinine	0.61	<0.001
	Takeaway food consumption	0.38	0.04
	FF section—food frequency (score) [38]	−0.37	0.01
Model 2	Creatinine	0.4	0.009
	Consumption of sauces or dressings in plastic containers	0.43	0.016
Model 3	Creatinine	0.36	0.014
Model 4	Creatinine	0.75	<0.001
	High-frequency use of canned (or tetra packed) food	−0.36	0.04

Legend. Model 1, BPA as the dependent variable; Model 2, MEP as the dependent variable; Model 3, MIbP as the dependent variable; Model 4, Factor 1 as the dependent variable.

**Table 7:** Multivariable analysis models (multivariable regression).

Dependent Variable	Predictor Factor	Standardized Coefficients		Not Standardized Coefficients		95% CI
		β	*p*	B	SE	
BPA	Creatinine	0.63	<0.001	0.36	0.09	0.18–0.548
	Takeaway cooked food consumption	0.27	0.06	0.14	0.07	−0.01–0.28
MEP	Creatinine	0.35	0.06	0.50	0.25	−0.013–1.00
	Consumption of sauces or dressings in plastic containers	0.37	0.037	0.39	0.18	0.03–0.74
Factor 1	Creatinine	0.71	<0.001	2.07	0.41	1.23–2.90

Legend. BPA, bisphenol A; MEP, monoethyl phthalate; SE, standard error; 95% CI, 95% confidence interval. Covariates included in the models. BPA: creatinine, takeaway cooked food consumption and FF section—food frequency; MEP: creatinine and sauces or dressings in plastic containers consumption; Factor1: creatinine and high-frequency use of canned (or tetra packed) food.

## Data Availability

All data presented in this study, not yet publicly archived, shall be made available through the corresponding author on request.

## Supplementary Materials

The following are available online at https://www.mdpi.com/article/10.3390/ijerph18189710/s1, Table S1: Urinary unadjusted concentrations of BPA and phthalates from literature in pregnant or non-pregnant women of childbearing age.
