# Supplementary material for: Lifestyle Habits and Exposure to BPA and Phthalates in Women of Childbearing Age from Northern Italy: A Pilot Study
Source: Int J Environ Res Public Health. 2021 Sep 15;18(18):9710. doi: 10.3390/ijerph18189710 (PMC8469822; doi:10.3390/ijerph18189710)
Supplement: Supplementary file 1 [file ijerph-18-09710-s001.zip › ijerph-1342419-supplementary.pdf]

Table S1. Urinary unadjusted concentrations of BPA and phthalates from literature in childbearing age women, either non-pregnant [59, 60, 63, 64] or pregnant [59, 62].

BPA (µg/l)<br>Median [95%CI]	*MEP (ng/ml)*<br>Median [Q1, Q3]<br>or **GM [95%CI]**	*MIbP (ng/ml)*<br>Median [Q1, Q3]<br>or **GM [95%CI]**	*MEHHP (ng/ml)*<br>Median [Q1, Q3]<br>or **GM [95%CI]**	*MEHP (ng/ml)*<br>Median [Q1, Q3]<br>or **GM [95%CI]**	*MBzP (ng/ml)*<br>Median [Q1, Q3]<br>or **GM [95%CI]**
**1.91**<br>**[1.3-2.5]**<br>[63]	**138**<br>**[108-176]**<br>[59]	**9.3**<br>**[8.3-10.5]**<br>[62]	**23**<br>**[20.1-26.3]**<br>[62]	**5.1**<br>**[4.3-6]**<br>[62]	**9.3**<br>**[8.3-10.5]**<br>[62]
	47.1<br>[20, 132]<br>[60]	**2.3**<br>**[1.92-2.8]**<br>[59]	**22.1**<br>**[18.8-26]**<br>[59]	**6.6**<br>**[5.7-7.5]**<br>[59]	**3.5**<br>**[2.9-4.3]**<br>[59]
	**152.9**<br>**[135.7-172.3]**<br>[64]	19.7<br>[12, 34]<br>[60]	**22.9**<br>**[20.3-25.9]**<br>[64]	2.07<br>[1.0, 3.7]<br>[60]	5.84<br>[3.3, 11]<br>[60]
		**4.3**<br>**[4.0-4.7]**<br>[64]	9.12<br>[6.2, 14]<br>[60]	**3.5**<br>**[3.2-3.9]**<br>[64]	**9.8**<br>**[8.9-10.8]**<br>[64]

Legend. In bold GM (Geometric Mean) and 95%CI (95% Confidence Interval).
